# Assessment of neuro-pulmonary crosstalk in asthmatic mice: effects of DiNP exposure on cellular respiration, mitochondrial oxidative status and apoptotic signaling

**DOI:** 10.1038/s41598-024-65356-y

**Published:** 2024-06-26

**Authors:** Samuel Abiodun Kehinde, Abosede Temitope Olajide, Ayokanmi Ore, Sarva Mangala Praveena, Farid S. Ataya, Ahmed M. El-Gazzar

**Affiliations:** 1 Biochemical Toxicology Laboratory, Faculty of Basic Medical Sciences, Ajayi Crowther University, Oyo, Nigeria; 2 Cell and Signaling Laboratory, Department of Biomedical Science, Faculty of Medicine and Health Sciences, Universiti Putra Malaysia (UPM), 43400 Serdang, Selangor Darul Ehsan Malaysia; 3 Redox Biochemistry, Metabolic and Phytotherapy Research Laboratory, Department of Chemical Sciences, Faculty of Natural Science, Ajayi Crowther University, Oyo, Nigeria; 4 Department of Environmental and Occupational Health, Faculty of Medicine and Health Sciences, Universiti Putra Malaysia (UPM), 43400 Serdang, Selangor Darul Ehsan Malaysia; 5 Department of Biochemistry, College of Science, King Saud University, PO Box 2455, 11451 Riyadh, Saudi Arabia; 6 Department of Experimental Pathology and Tumor Biology, Nagoya City University Graduate School of Medical Sciences, Nagoya, Japan

**Keywords:** Diisononyl phthalates, Asthma, Brain, Cellular respiration, Mitochondria, Apoptosis, Biochemistry, Cancer, Cell biology, Chemical biology, Developmental biology, Neuroscience, Biomarkers, Diseases, Neurology, Oncology

## Abstract

Human health is becoming concerned about exposure to endocrine disrupting chemicals (EDCs) emanating from plastic, such as phthalates, which are industrially employed as plasticizers in the manufacturing of plastic products. Due to some toxicity concerns, di(2-ethylhexyl) phthalate (DEHP) was replaced by diisononyl phthalate (DiNP). Recent data, however, highlights the potential of DiNP to interfere with the endocrine system and influence allergic responses. Asthma affects brain function through hypoxia, systemic inflammation, oxidative stress, and sleep disturbances and its effective management is crucial for maintaining respiratory and brain health. Therefore, in DiNP-induced asthmatic mice, this study investigated possible crosstalk between the lungs and the brain inducing perturbations in neural mitochondrial antioxidant status, inflammation biomarkers, energy metabolizing enzymes, and apoptotic indicators. To achieve this, twelve (n = 12, 20–30 g) male BALB/c mice were divided into two (2) experimental groups, each with five (6) mice. Mice in group II were subjected to 50 mg/kg body weight (BW) DiNP (Intraperitoneal and intranasal), while group I served as the control group for 24 days. The effects of DiNP on neural energy metabolizing enzymes (Hexokinase, Aldolase, NADase, Lactate dehydrogenase, Complex I, II, II & IV), biomarkers of inflammation (Nitric oxide, Myeloperoxidase), oxidative stress (malondialdehyde), antioxidants (catalase, glutathione-S-transferase, and reduced glutathione), oncogenic and apoptotic factors (p53, K-ras, Bcl, etc.), and brain histopathology were investigated. DiNP-induced asthmatic mice have significantly (*p* < 0.05) altered neural energy metabolizing capacities due to disruption of activities of enzymes of glycolytic and oxidative phosphorylation. Other responses include significant inflammation, oxidative distress, decreased antioxidant status, altered oncogenic-apoptotic factors level and neural degeneration (as shown in hematoxylin and eosin-stained brain sections) relative to control. Current findings suggest that neural histoarchitecture, energy metabolizing potentials, inflammation, oncogenic and apoptotic factors, and mitochondrial antioxidant status may be impaired and altered in DiNP-induced asthmatic mice suggesting a pivotal crosstalk between the two intricate organs (lungs and brain).

## Introduction

Diisononyl phthalate (DINP) is a plasticizer commonly utilized in various plastic items. It has been associated with various health effects. DINP is mainly used as a plasticizer to confer flexibility in polyvinyl chloride (PVC)-based products and its common applications include personal care items, nutritional supplements, and pharmaceuticals.

DiNP exposure has been associated with different forms of physiological impairment such as oxidative stress, leading to the depletion of antioxidants such as reduced glutathione and ascorbic acid, while increasing levels of malondialdehyde (a marker of oxidative damage)^1,2^. In the brain, oxidative stress disrupts cellular homeostasis, damages biomolecules, and contributes to neurodegenerative conditions. Furthermore, DiNP-treated mice from a previous study showed elevated levels of inflammation biomarkers such as myeloperoxidase and nitric oxide. Chronic inflammation can impair brain function, disrupt neural networks, and contribute to neuroinflammatory diseases^3^.

The lungs and brain are metabolically linked through the cardiovascular system, where the exchange of gases and the maintenance of homeostasis are critical for the proper function of both organs. This interdependence ensures that the brain receives a constant supply of oxygen and that metabolic waste products are efficiently removed, thereby supporting optimal neurological and respiratory health^4^. Asthma can adversely affect brain function through a combination of hypoxia, systemic and neuroinflammation, psychological stress, sleep disturbances, and oxidative stress. These factors collectively contribute to cognitive impairments, mood disorders, and an overall decline in neurological health. Managing asthma effectively and addressing these associated factors is crucial for maintaining both respiratory and brain health^5^.

Exposure to DiNP has been associated with the aggravation of airway remodelling and airway hyperresponsiveness (AhR) in individuals progressing from atopic dermatitis (AD) to asthma. It induces an increase in interleukin-33 (IL-33), immunoglobulin E (IgE), Th2 and Th17 cytokines, and the expression of thymic stromal lymphopoietin (TSLP). Longitudinal studies in asthmatic children have shown that exposure to DINP is associated with increased fractional exhaled nitric oxide (FeNO) levels^6^. Other toxic effects includes eye and skin irritation, drowsiness and dizziness, fertility concerns and prolonged or repeated exposure to DINP can cause damage to organs.

DiNP has been established to induce energy metabolism impairment which was evident in the downregulated glycolytic enzymes and impaired oxidative phosphorylation enzymes^7^. In the brain, energy metabolism is crucial for neuronal function. Disruption of energy transduction can impact brain health^8^. Also, a study confirmed that DiNP exposure causes alterations in lung oncogenic markers, including increased expression of p53, Bax, c-MYC, and K-Ras. While this study focused on lung tissue, chronic exposure to DiNP may potentially affect brain cells similarly^2^. However, direct evidence linking DiNP to neural oxidative stress, inflammation, impaired energy metabolism, and expression of apoptotic markers remains limited. Hence, this study investigated possible crosstalk between the lungs and the brain causing perturbations in neural mitochondrial antioxidant status, inflammation biomarkers, energy transduction enzymes, and apoptotic indicators in DiNP-induced asthmatic mice.

## Materials and methods

### Assay kits and chemicals

DiNP was procured from Relonchem Ltd., Chesire, UK, Reduced glutathione is a product of Ak Scientific Inc, CA. CYPRESS Diagnostics, Belgium manufactured the Lactate Dehydrogenase test kit. Merck, Darmstadt, Germany supplied trichloroacetic acid and 1-chloro,2,4-dinitrobenzene, 5,5-dithio-bis-2-nitrobenzoic acid, thiobarbituric acid. The enzyme-linked immunosorbent assay (ELISA) kits for K-Ras, Caspase-3, c-Myc, Bcl-2, p53, and Bax were sourced from the Cusabio Technology Llc, TX, USA. Other analytical grade reagents and chemicals were sourced from Sigma Chemical Co., USA, and Carlroth GMBH, Karlsruhe, Germany.

### Experimental animals

This study utilized twelve (12) healthy male mice of 20–30 g in weight. A one-week acclimatization period at the animal facility unit of the Department of Chemical Sciences of Ajayi Crowther University, Nigeria preceded the commencement of the study, during which the mice were given standard laboratory chow and unlimited access to drinking water.

### Study design

The 50 mg/kg/day DiNP dosage was selected based on earlier research by Hwang et al^9^. DiNP was constituted in saline. Male mice (8 weeks old, 20 ± 10 g) were split into two (2) groups of six (6) mice each: I-control group (saline), and II- DiNP group (50 mg/kg BW—intranasal and intraperitoneal). The 23-day administration period was in effect. The Faculty of Natural Sciences at Ajayi Crowther University's Ethical Review Committee approved the experimental protocol under the reference number FNS/ERC/23/005AP. The mice received treatment in adherence to established guidelines for the care and handling of laboratory animals^10^. All mice were euthanized 24 h after the final administration.

### Induction of asthma

**Figure 1:**
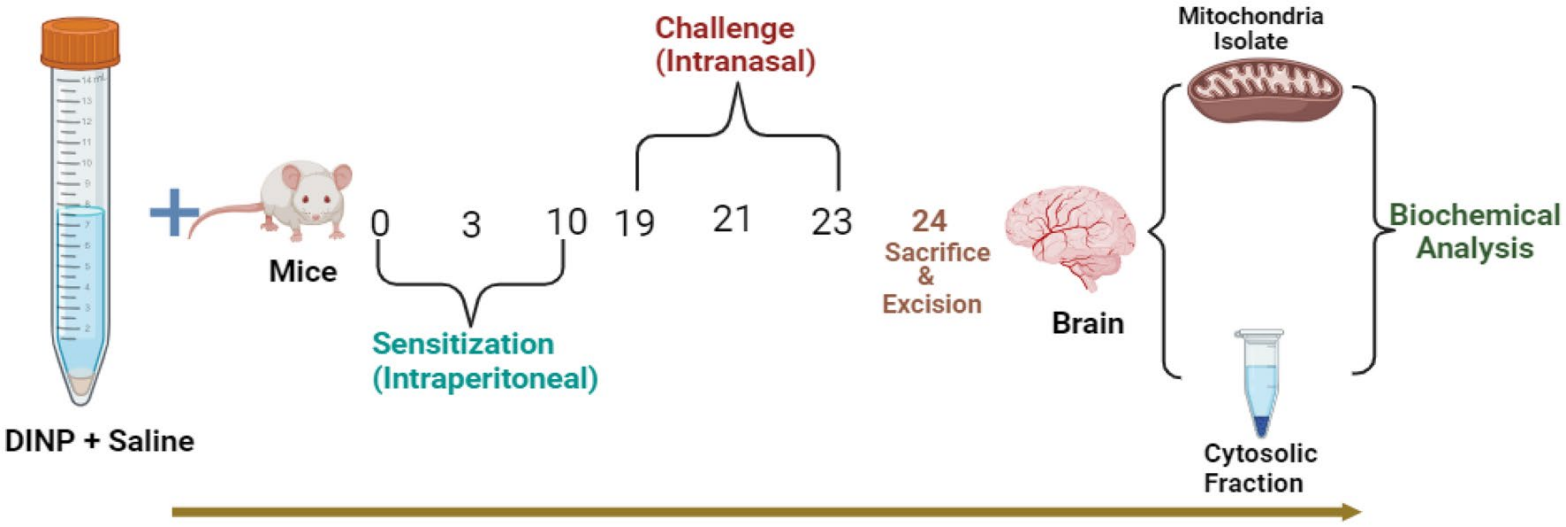
Experimental Design for DiNP-induced asthma (Created in BioRender.com).

Induction of asthma is as illustrated in Fig. 1. The primary method employed for sensitizing the mice involved an intraperitoneal injection of DiNP (50 mg/kg) on day 0 (Fig. 1). Secondary sensitization of the mice occurred through intraperitoneal injections of DiNP (50 mg/kg) on days 3 and 10. Furthermore, the mice received intranasal injections of 50 mg/kg of DiNP diluted in 50 mL of saline solution on days 19, 21, and 23. DiNP was administered intraperitoneally to sensitize the mice (Sensitization phase), intranasal administration of the DiNP was employed to challenge (Challenge phase).

### Collection of samples and Brain mitochondria isolation

When the mice were euthanized, the brain section (about 0.5 g) was removed, and it was homogenized using homogenization medium (0.5 M TRIS–HCl, 2 M EDTA, 5w/v: sucrose, pH 7.4) before being centrifuged for five minutes at 3000 rpm. After transferring the supernatant to 5 mL Eppendorf tubes, the mixture was centrifuged for two minutes at 13,000 rpm. A 2.0 mL Eppendorf tube was pre-weighed, the pellet containing the mitochondria was added, and the tube was centrifuged for two minutes at 13,000 rpm using homogenization media. Pellets were resuspended in MAITE medium (orthophosphoric acid, sorbitol, KCL, sucrose, 1 M MgCL_2_, 0.5 M EDTA, 0.5 M Tris-KCl, pH 7.4) after the supernatant was decanted. Following centrifugation for two minutes at 13,000 rpm, the supernatant was poured off. Subsequently, the 2 mL Eppendorf tube containing the mitochondria was reweighed, and the weight of the empty tube was subtracted from the weight of the tube containing the isolated mitochondria. This yielded the weight of the mitochondria. Re-suspended in 1 mL of MAITE media, the mitochondrial isolate was then preserved. All centrifugation and homogenization processes were conducted at 4 °C.

### Neural antioxidant and oxidative stress biomarkers

The level of reduced glutathione (GSH) was assessed in accordance with Rahman et al.^11^ Catalase (CAT) activity was measured using Hadwan and Abed's^12^ methodology. Malondialdehyde concentration (MDA) was estimated following the protocol of Tsikas et al.^13^, and the brain glutathione S-transferase (GST) activity and lipid peroxidation marker were estimated using the Habig et al.^14^ and Vashney and Kales^15^ methods respectively.

### Neural inflammation

The concentration of nitric oxide (NO) in the brain sample was determined using Bryan et al.^16^. The method outlined by Kim^17^ was utilized to spectrophotometrically assess the activity of myeloperoxidase (MPO).

### Neural glycolytic enzymes

The assessment of hexokinase activity was carried out using the procedure described by Colowick^18^. NADase activity was estimated for with a procedure described by Kim et al.^19^. Jagannathan et al.^20^ established protocol was utilized to evaluate aldolase catalytic activity. LDH activity was determined using the LDH Kit in accordance with the instruction of the manufacturer.

### Neural tricarboxylic acid cycle enzymes

Using the spectrophotometric enzyme protocols outlined by Yu et al.^21^, citrate synthase (CS) activity was evaluated. As Romkina and Kiriukhin^22^ previously outlined, the isocitrate dehydrogenase (IDH) activity was determined. The method described by Thorne et al.^23^ was used for evaluating the activity of malate dehydrogenase (MDH). The method outlined by Jones and Hirst^24^ was used to assess the activity of succinate dehydrogenase (SDH) spectrophotometrically.

#### Neural electron transport chain enzymes

The respiratory complexes (I, II, III, and IV) activities were also spectrophotometrically determined at 340 nm in the mitochondria, using the protocol detailed by Medja et al.^25^.

#### Neural oncogenic and apoptotic (Bax, c-Myc, Bcl-2, Ras, p53, and caspase-3) factors

The Cusabio Technology Llc, TX, USA, ELISA kits' instructions were adhered, to assess the concentration of c-Myc, caspase-3, Bax, p53, Ras and Bcl-2.

#### Total Protein Determination:

The amount of total protein in spleen and lymphocytes was measured using the method of Gornall, et al.^26^.

#### Neural histopathology

Fischer et al.^27^ outline for preparing neural tissue sections for hematoxylin and Eosin (H & E) staining was used. A 5-µm thin section of brain was cut on a microscope slide, immersed for thirty seconds in distilled water (dH_2_O), followed by thirty sections in hematoxylin, and then rinsed for one minute with dH_2_O. After staining the slides with eosin, they were dehydrated in 95% alcohol and then in 100% alcohol for 30 s each. After extracting the alcohol from the slides using xylene, two drops of glycerol (a mounting medium) were added, and the slides were covered with a coverslip for microscopic inspection. A pathologist conducted the histological analysis and provided the explanations.

#### Statistical analysis

Data from this study were normally distributed and T-test was used to compare the means of the control group and DiNP-treated group. Statistical (T-test) and graphical presentation was done using Prism 8.0.1(GraphPad Software Inc., USA) and data are expressed as mean ± standard deviation (SD) of five replicates. The data underwent analysis where statistical significance was based on p values less than 0.05.

## Results

### DiNP-induced asthma stimulates neural oxidative stress

**Figure 2:**
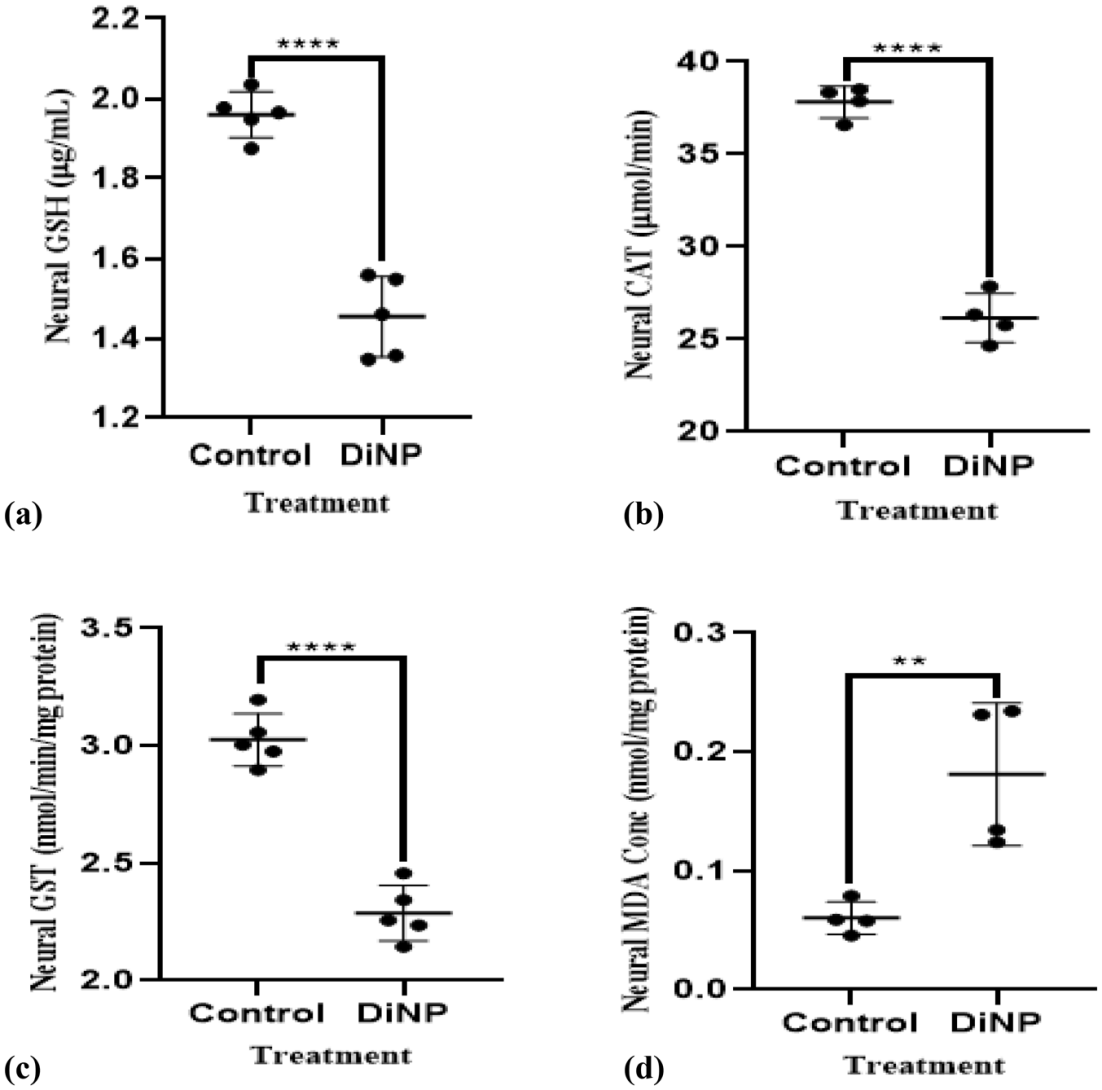
Neural antioxidant enzyme activities in DiNP-induced asthmatic mice. The values represent the mean ± SD. (n = 5) per group. ∗  = significantly (*P* ˂0.05) different relative to control. GSH = Glutathione, MDA = Malondialdehyde, GST = Glutathione-s-transferase, CAT = Catalase.

Figure 2, shows DiNP-induced asthmatic mice group had a significantly higher level of neural MDA (38.89%) than the control group. The brains of mice given 50 mg/kg DiNP revealed a significant decline in GSH (76.31%) level and GST (75.02%) and CAT (69.33%) activities compared to the control.

### DiNP-induced asthma elicited neural inflammation

**Figure 3:**
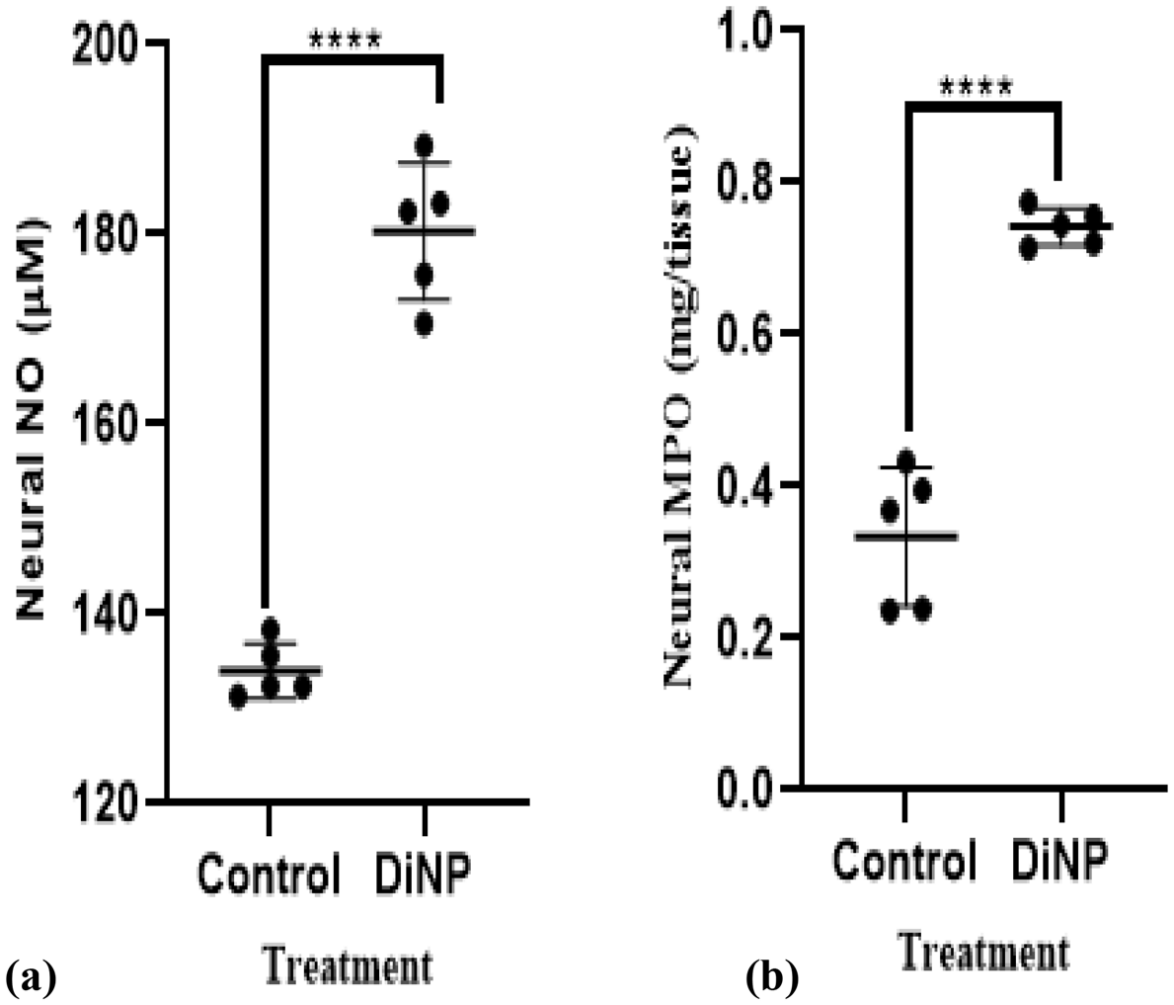
Neural cardiac inflammatory biomarkers in DiNP-induced asthmatic mice. The values represent the mean ± SD. (n = 5) per group. ∗  = significantly (*P* ˂0.05) different relative to control. NO = Nitric oxide, MPO = Myeloperoxidase.

Figure 3 illustrates the perturbations in level of neural nitric oxide (NO) and the activity of myeloperoxidase (MPO) orchestrated by DiNP-induced asthma. There was a significant (*p* < 0.05) elevation in neural concentration of NO (26.33%) and MPO activity (41.02%) in mice administered DiNP relative with the control.

### DiNP-induced asthma perturbs neural glycolytic enzymes activities in mice

**Figure 4:**
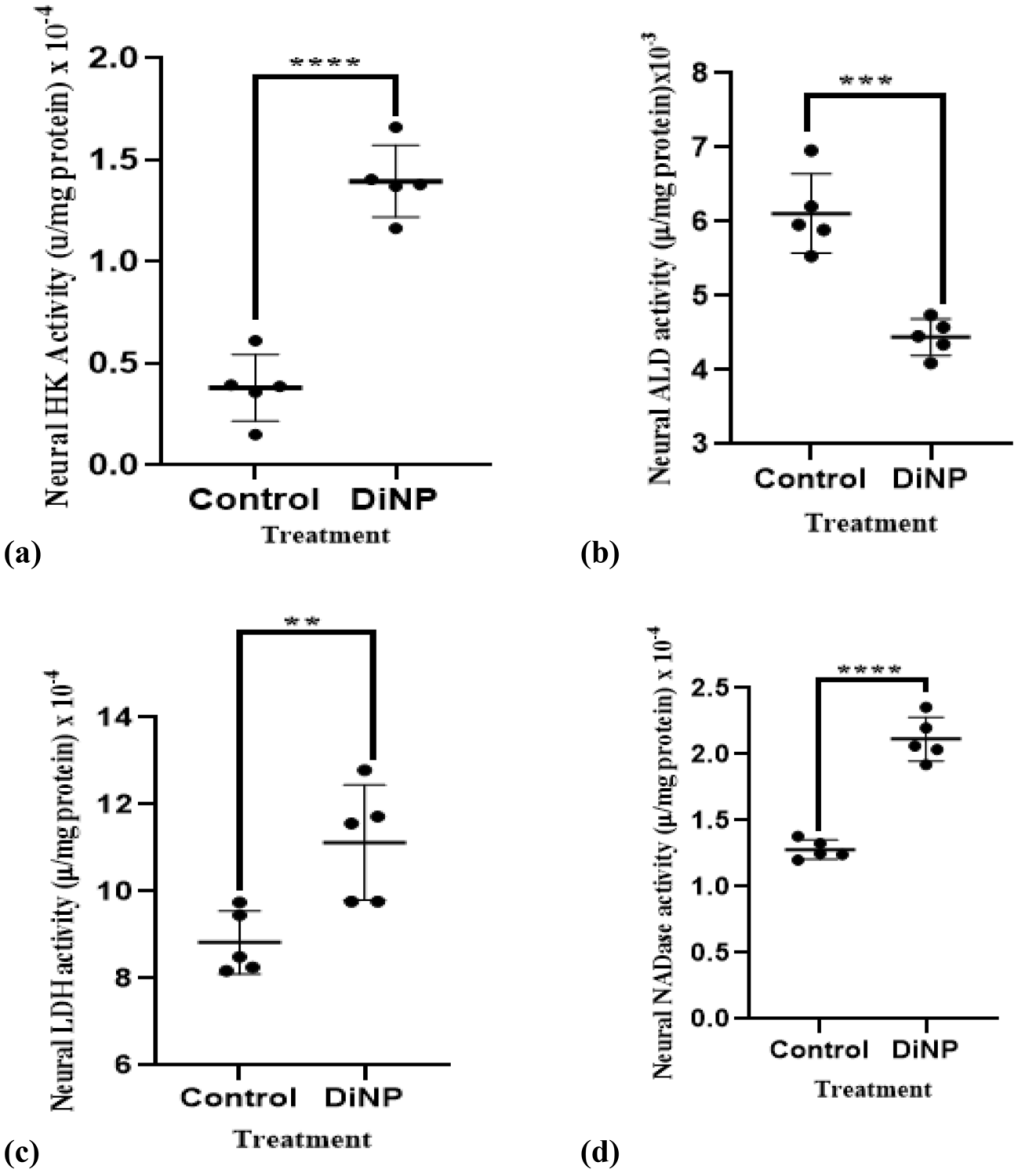
Neural glycolytic enzyme activities in DiNP-induced asthmatic mice. The values represent the mean ± SD. (n = 5) per group. ∗  = significantly (*P* ˂0.05) different relative to control. HK = Hexokinase, LDH = Lactate dehydrogenase, ALD = Aldolase, NADase = NAD^+^ Nucleosidase.

The effect of DiNP-induced asthma on neural enzymes activities of glycolysis is shown in Fig. 4. Relative to the control group, DiNP significantly (*p* < 0.05) up-regulated the activities of HK (71.01%), LDH (22%), NADase (59.52%) by while a downregulated activity of ALD (26.22%) was observed.

### DiNP-induced Asthma alters neural tricarboxylic acid cycle enzymes activities

**Figure 5:**
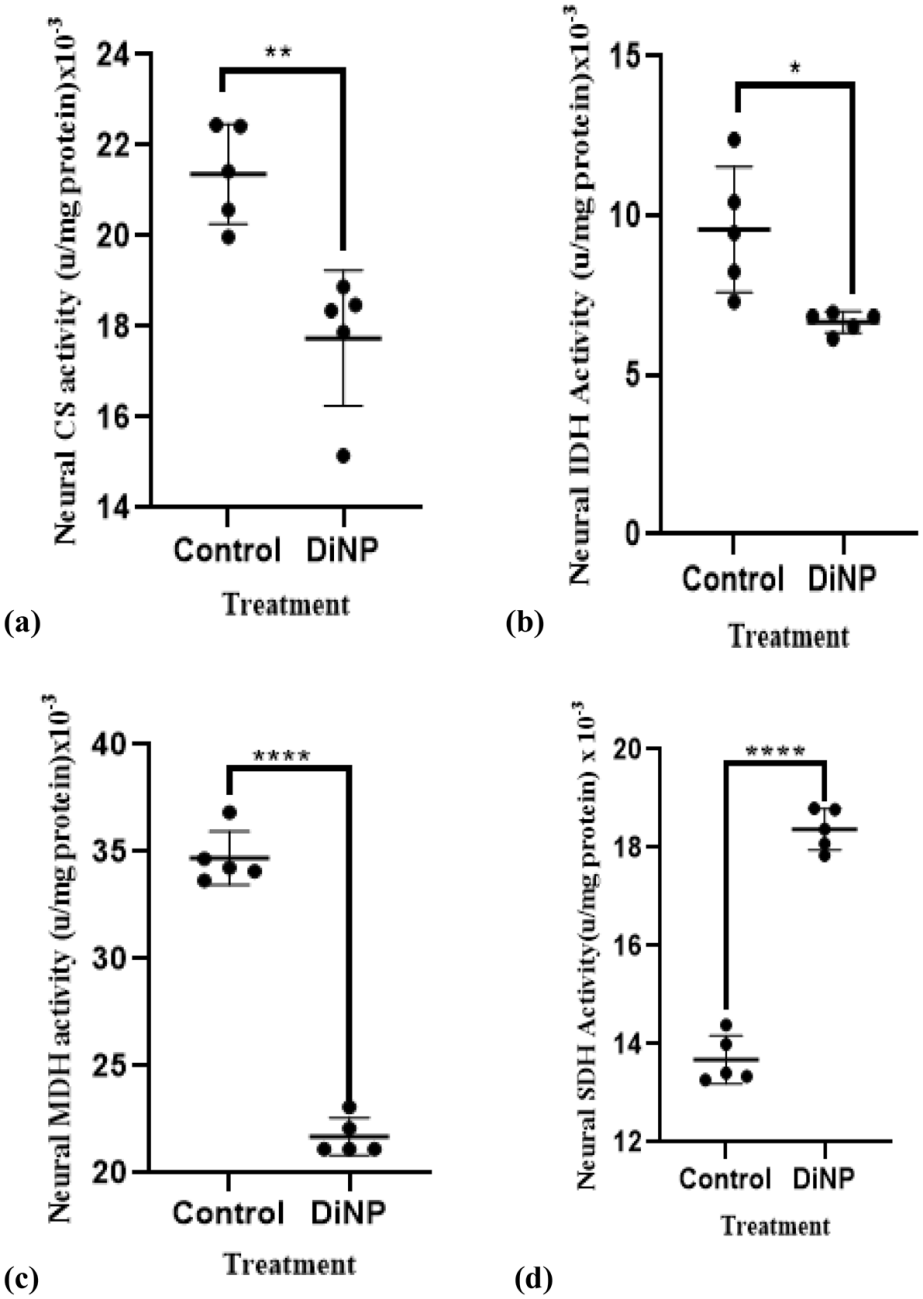
Neural tricarboxylic acid cycle enzyme activities in mice. The values represent the mean ± SD. (n = 5) per group. ∗  = significantly (*P* ˂0.05) different relative to control. SDH = Succinate dehydrogenase, MDH = Malate dehydrogenase, Isocitrate Dehydrogenase = IDH, Citrate synthase = CS.

Figure 5 depicts DiNP-induced variations in tricarboxylic acid cycle enzyme activity. DiNP-induced asthma significantly (*p* < 0.05) decreased the activities of neural CS (17.25%), IDH (22.22%), MDH (37.68%), and increased SDH (27.02%) activity compared to the control group.

### DiNP-induced asthma alters neural activities of electron transport chain enzymes in mice

**Figure 6:**
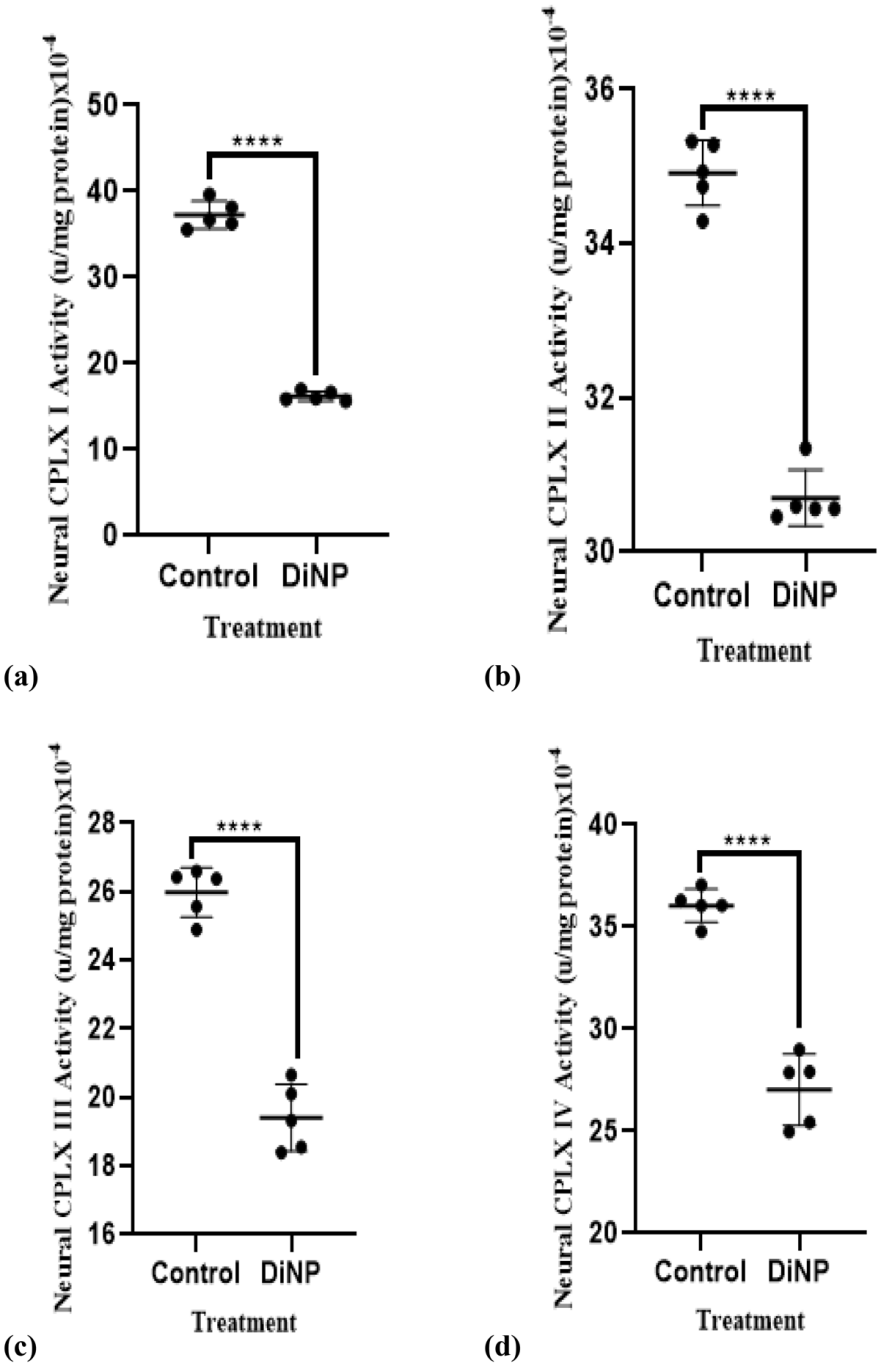
Neural respiratory chain enzyme activities in mice. The values represent the mean ± SD. (n = 5) per group. ∗  = significantly (*P* ˂0.05) different relative to control. Complex I = (CPLX I), Complex II = (CPLX II), Complex III = (CPLX III), Complex IV = (CPLX IV).

Figure 6 depicts DiNP-induced variations in the activity of electron transport chain enzymes. DiNP significantly (*p* < 0.05) lowered CPLX I, II, III, and IV (60.40%, 87.14%, 73.07% and 27.49% respectively) activity compared to control group.

### DiNP-induced asthma alters neural oncogenic biomarkers and apoptotic factors in mice

**Figure 7:**
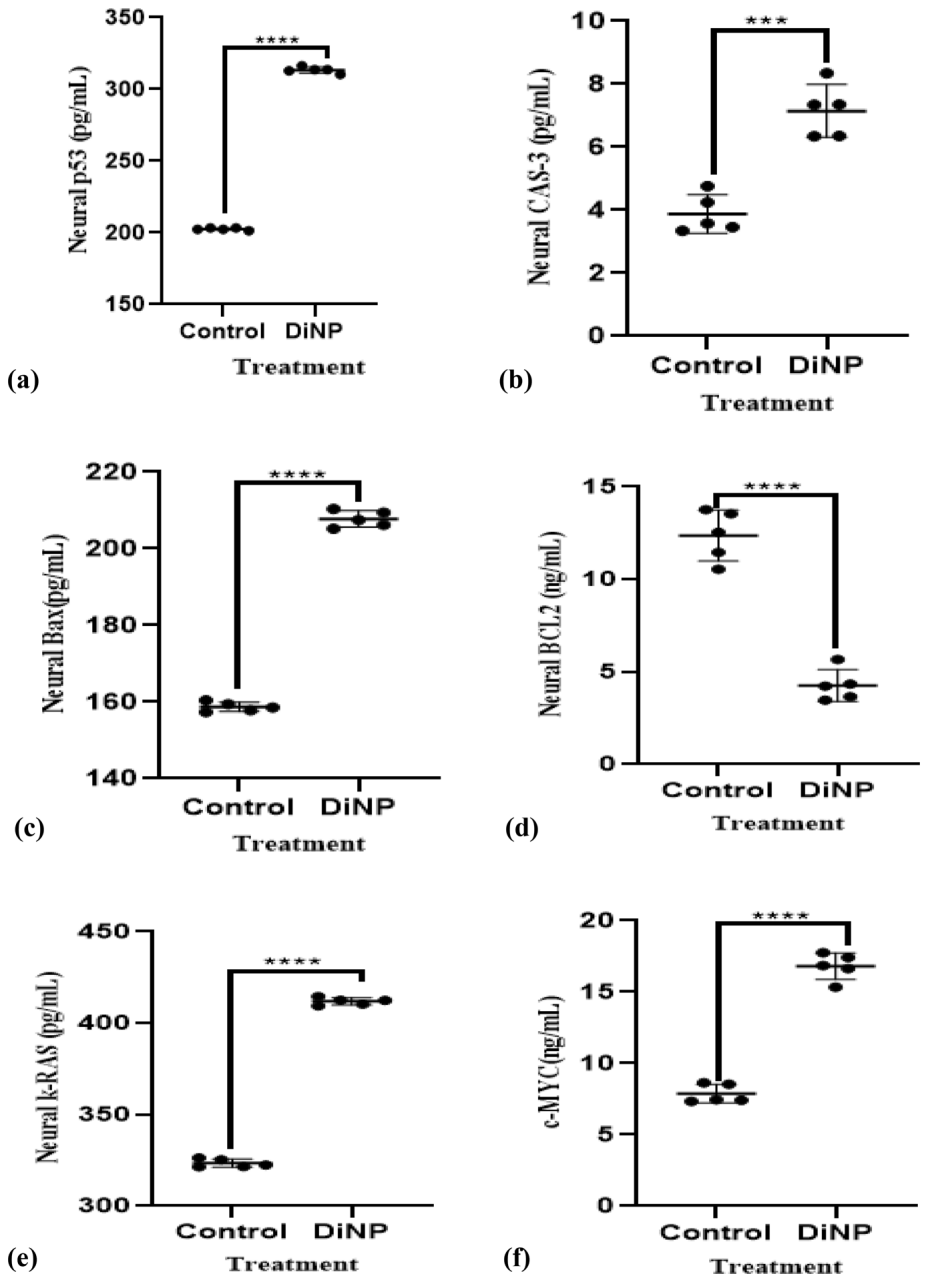
Neural oncogenic and apoptotic factors in DiNP-induced asthmatic mice. The values represent the mean ± SD. (n = 5) per group. ∗  = significantly (*P* ˂0.05) different relative to control. Cas-3 = Caspase-3, K-Ras = Kirsten rat sarcoma virus, p53 = Tumor protein P53 factors, Bax = Apoptosis regulator BAX, Bcl-2 = B-cell lymphoma 2, c-MYC = Cellular Myelocytomatosis.

DiNP-induced asthma causes changes in neural oncogenic markers and apoptotic factors, as shown in Fig. 7. Specifically, DiNP-induced asthma significantly (*p* < 0.05) decreases neural Bcl-2 levels by 64.34% and increases the levels of Cas-3, p53, Bax, K-Ras and c-MYC (57.14, 64.52, 76.19, 23.81 and 53.12% respectively) compared to the control group.

### Distortion of neural histoarchitecture in DiNP-induced asthmatic mice

**Figure 8:**
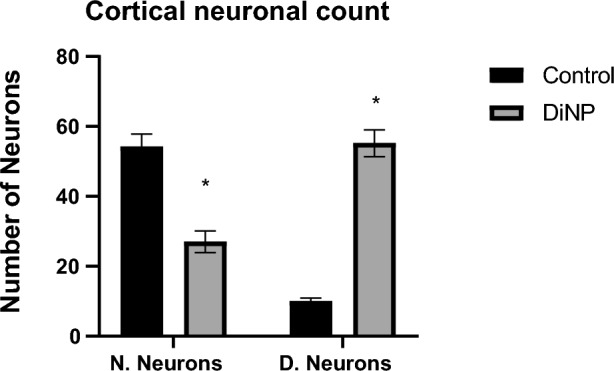
Bar chart showing the neuronal count of the prefrontal cortex of the brain. *—significant difference when DiNP group (experimental) is compared with control. N. Neurons—Normal Neurons, D. Neurons—Neurons showing degenerating features. Values are expressed as mean ± SD.

Using the Image J software Cell Counter tool, cortical neuronal count of prefrontal cortex was evaluated. In this investigation, there was a significant (*p* < 0.0001) increase in degenerating neuronal count of the prefrontal cortex in the experimental (DiNP-induced asthmatic group) group relative with control group (Fig. 8). Also, there was a significant (*p* < 0.0001) decrease in normal neuronal count when experimental group was compared with control group.

## Discussion

Endocrine-disrupting chemicals (EDCs) such as phthalates, is an exogenous substance that interfere with hormone action in the body and has been shown to block, mimic, or alter the function of natural hormones, leading to various health effects. EDCs has the potential to induce oxidative stress, cause fertility and developmental problems, hormone-sensitive cancer, obesogenic effects, and thyroid and adrenal interference Etc^28,29^. This study examined the crosstalk effect of DiNP, a class of the EDCs, on neural mitochondrial oxidative stress, inflammation, impaired energy metabolism, and apoptotic factors in DiNP-induced asthmatic mice.

Various EDCs, including phthalates, have been associated with oxidative stress and the mechanisms include generation of reactive oxygen species (ROS), disruption of antioxidant systems, and mitochondrial dysfunction. Oxidative stress induced by EDCs can impact endocrine organs (e.g., thyroid, adrenal glands, and gonads) and other tissues, it may also contribute to health conditions such as infertility, metabolic disorders, and cancer^28^. Relative to the control group, a decrease in the concentration and activities of neural antioxidants (such as the CAT, GSH and GST), and an increase in malondialdehyde (MDA, a marker of lipid peroxidation due to oxidative stress) level was observed in the DiNP-induced asthmatic mice. Hence, this suggests that DiNP successfully induce oxidative stress in the brain of the asthmatic mice by disrupting the antioxidant system (observed reduced concentration and activity of GSH, CAT and GST and increased level of MDA).

Myeloperoxidase (MPO) and Nitric oxide (NO) has been shown to have a co-interaction in inflammatory response. MPO's catalytic activity is influenced by its affinity for NO versus hydrogen peroxide (H_2_O_2_) and the amounts of both^30^. Under ideal physiological conditions, NO acts as an anti-inflammatory agent while on the other side, is regarded as a pro-inflammatory mediator that induces inflammation when generated in excess under abnormal situations, whereas MPO (which is released by neutrophils and monocytes as a component of the innate immune system) catalyses the formation of hypochlorous acid (HOCl), a powerful oxidant. However, in a normal condition, HOCl is involved in microbial killing during infection, but its overproduction may eventually result into inflammation^31,32^. In this study, an elevated level of NO, and MPO activity were observed in the brain of DiNP- asthmatic mice, thus, justifying the ability of DiNP to induce neural inflammation via the overproduction of H_2_O_2_ and HOCl.

Furthermore, the brain primarily relies on glucose as its primary energy substrate, and it is a simple sugar that serves as the obligatory fuel for brain cells. The human brain utilizes around 20% of the energy derived from glucose. Neurons, the functional units of the brain, are highly dependent on glucose for their energy needs. Unlike other organs, the brain has limited capacity to store glucose, so it relies on a continuous supply from the bloodstream. Brain cells primarily use the glycolytic pathway to break down glucose into pyruvate, however, during fasting or prolonged exercise, the brain can also use ketone bodies (such as β-hydroxybutyrate) as an alternative energy source^33^. Phthalates have been associated with disruptions in energy metabolism. Some phthalates can disrupt thyroid function, the gland that regulate metabolism, and any disruption can impact energy utilization. Phthalates have also been linked to insulin resistance and impaired glucose metabolism^34^. Also, mitochondria disruption is notably one of the effects of exposure to phthalate and this may affect mitochondrial function by disrupting energy generation^35^. The energy disruptive effects of DiNP were evident in the findings of this study as the activities of the key enzymes of the glycolytic pathway, tricarboxylic acid pathway and electron transport chain enzymes were altered in the neural cells of the DiNP-induced asthmatic mice. Some of the enzymes of the energy metabolism pathway HK, LDH, NADase and SDH was however found to have an increased activity. This may be due to what is referred to as compensatory mechanisms, because when certain metabolic pathways are disrupted (such as glycolysis), cells may activate compensatory mechanisms.

Hexokinase phosphorylates glucose to glucose-6-phosphate, initiating glycolysis and maintaining intracellular glucose levels, thus influencing metabolic flux. Aldolase cleaves fructose-1,6-bisphosphate into crucial intermediates for ATP and pyruvate production, essential for energy in muscle and liver tissues and supporting gluconeogenesis^36^. NADase hydrolyzes NAD + to nicotinamide and ADP-ribose, regulating NAD + levels for redox reactions, DNA repair, and cell survival, maintaining metabolic balance. Lactate Dehydrogenase (LDH) converts pyruvate to lactate under anaerobic conditions and vice versa, enabling glycolysis in hypoxia by regenerating NAD + . Elevated LDH levels indicate tissue damage and serve as diagnostic markers for diseases like myocardial infarction and liver disease, allowing cells to adapt to fluctuating oxygen levels^37^.

Increased NADase activity could be an adaptive response to maintain cellular homeostasis despite reduced glycolytic enzyme activity. Also, it may be due to redox balance and NAD + availability, NADase cleaves NAD^+^ into nicotinamide and ADP-ribose. Nicotinamide can be recycled to regenerate NAD^+^, the neural cells may prioritize maintaining NAD^+^ levels for redox reactions and other essential processes. Cellular stress and inflammation may be one of the contributing factors to the increased NADase activity, reduced glycolytic enzyme activity can lead to cellular stress. Inflammatory conditions or stress responses may trigger NADase activation^38–40^. Furthermore, the increased activities of HK, LDH, and SDH can be influenced by various factors and have different implications: increased HK activity may occur due to increased energy demand, to enhance glucose utilization, hormonal regulation, and cell-specific needs (that is, different tissues express specific hexokinase isozymes (e.g., glucokinase in the liver). Increased LDH activity may be associated with tissue damage because LDH is released during injury or disease, energy demand (LDH can increase during high energy requirements), pH changes, and it is also an indicator of metabolic shifts or tissue stress. SDH is involved in the TCA cycle and electron transport chain. Increased SDH activity may result from cellular respiration, regulation by factors like hormones and oxygen availability, and cell-specific needs as SDH operates differently in various tissues. Elevated SDH activity can impact cellular metabolism and energy production^41–44^.

Apoptosis is a crucial process of programmed cell death that is seen in malignant and healthy tissues. In healthy tissues, it induces damaged or mutated cells to die, which prevents further mutations and the growth of cancer. The result of this investigation showed a decreased BCL-2 and an increased CAS-3, Bax, p53, c-MYC, and k-Ras concentration in the neural cells of the DiNP-induced asthmatic mice. The observed decreased concentration of BCL-2 may be associated with a reduced ability of cells to inhibit apoptosis (programmed cell death), and this could potentially make cancer cells more susceptible to cell death. While the increased levels of p53, Cas-3, Bax, k-Ras and c-MYC are often linked to DNA damage or cellular stress, activation, and promotion of apoptosis. Bax is a pro-apoptotic protein that counteracts the anti-apoptotic effects of BCL-2. Notably, the elevated level of Bax must have inhibited the anti-apoptotic function of BCL-2 since a decreased level of BCL-2 was observed in the cellular cells of the DiNP-induced asthmatic mice. In addition, increased of K-Ras is often associated with cancer progression. K-Ras mutations can lead to uncontrolled cell growth and survival, while increased c-MYC expression is common in many cancers^45–48^. Given these observations, it is believed that DiNP may promote and be an active substance that can induce cancers in the brain.

**Figure 9:**
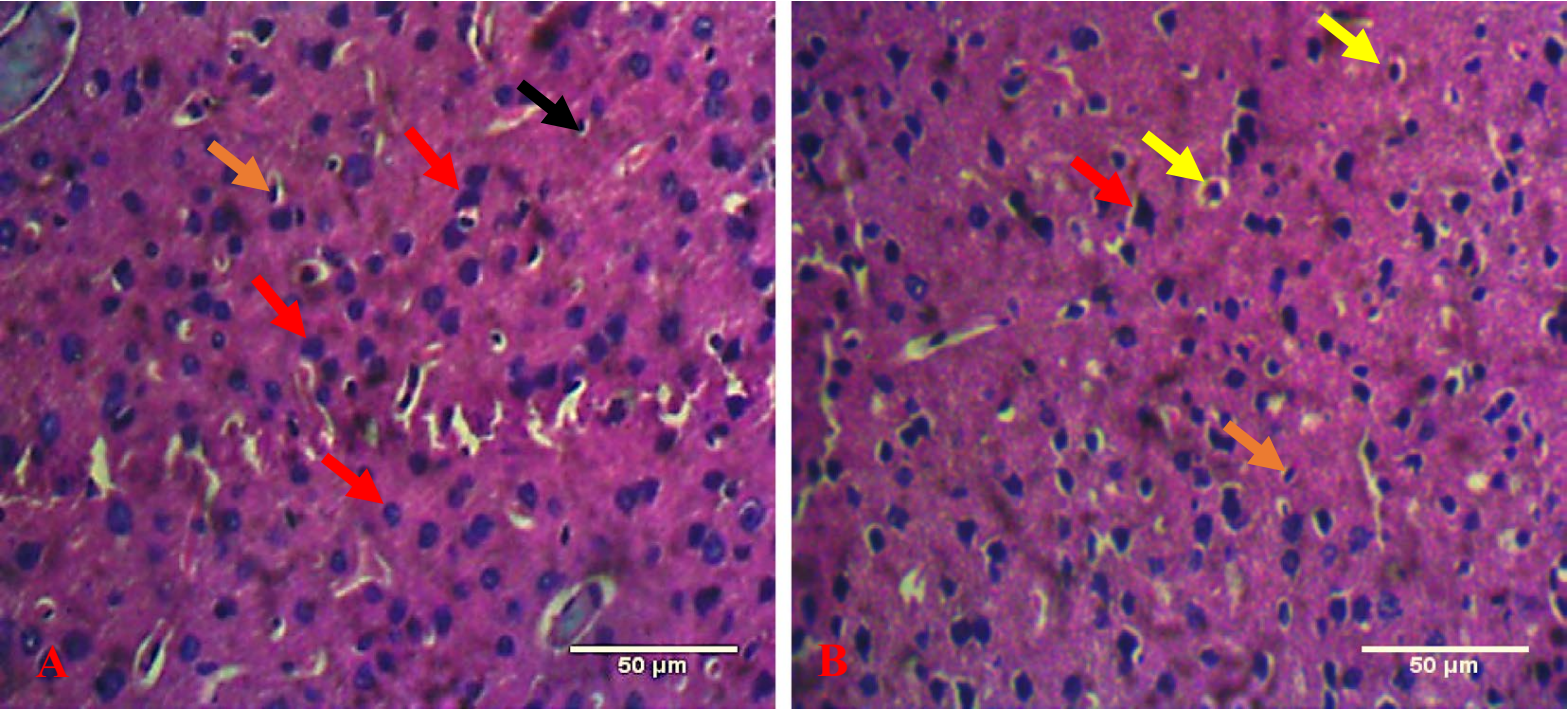
Representative photomicrographs of brain sections subjected to H&E stain. (**A**) Control group, (**B**) DiNP group. Red arrow—intact neuron, black arrow- Oligodendrocyte, yellow arrow- degenerating (vacuolated) neuron, orange arrow- Degenerating (pyknotic neuron respectively) Scale bar- 50 μm.

The histological analysis of the prefrontal cortex revealed that DiNP (diisononyl phthalate) neurotoxicity caused a variety of neuronal injuries. The affected neurons exhibited several distinct patterns of degeneration. These included acidophilic neurons characterized by shrunken, condensed, and pyknotic nuclei; eosinophilic neurons with slightly darker nuclei or without any identifiable nuclei; and neurons undergoing karyorrhexis, where the chromatin fragments, as shown in Fig. 9. Previous studies have also reported significant atrophy in the superficial layers of the sensorimotor cortex. This atrophy is often accompanied by dendritic sprouting, suggesting that the prefrontal cortex is undergoing injury, reorganization, and neuroplastic changes within the neocortical networks in response to the damage^49^. These histological changes were predominately observed in the DiNP only group which points to the neurotoxic effect of DiNP to initiate such neuronal damage in the prefrontal cortex.

## Conclusion

Complex pathways exist between the brain and the lungs, and abnormalities in lung function can in fact impact the health of the brain. The brain-lung axis is still an emerging area of investigation, and the current findings from this study suggest that in a DiNP-asthmatic mice, depletion of lung functions due to the asthmatic conditions of the mice can perturb neural mitochondrial antioxidant status, inflammation biomarkers, energy metabolizing enzymes, and apoptotic indicators. The alterations observed in the energy metabolizing enzymes evident in an increased activities of some of the enzymes may reflect adaptations of the brain cells to changing conditions or metabolic needs during episodes of asthma being induced by DiNP.

## Data Availability

The data generated during and/or analyzed during the current study can be requested from the corresponding author upon reasoned request.
